# Synergistic Anticancer Activities of Natural Substances in Human Hepatocellular Carcinoma

**DOI:** 10.3390/diseases3040260

**Published:** 2015-10-22

**Authors:** Akiko Kojima-Yuasa, Xuedan Huang, Isao Matsui-Yuasa

**Affiliations:** 1 Department of Food and Human Health Sciences, Graduate School of Human Life Science, Osaka City University, 3-3-138 Sugimoto, Sumiyoshi-ku, Osaka 558-8585, Japan; E-Mail: yuasa-i@hotmail.co.jp; 2 Department of Pharmacognosy, School of Pharmacy, Kitasato University, 5-9-1 Shirogane, Minato-ku, Tokyo 108-8641, Japan; E-Mail: kouse@platinum.pharm.kitasato-u.ac.jp

**Keywords:** hepatocellular carcinoma (HCC), natural products, chemotherapeutic agents, combination treatment, synergistic anticancer effect, reactive oxygen species (ROS)

## Abstract

Hepatocellular carcinoma (HCC) is highly resistant to currently available chemotherapeutic agents. The clinical outcome of HCC treatment remains unsatisfactory. Therefore, new effective and well-tolerated therapy strategies are needed. Natural products are excellent sources for the development of new medications for disease treatment. Recently, we and other researchers have suggested that the combined effect of natural products may improve the effect of chemotherapy treatments against the proliferation of cancer cells. In addition, many combination treatments with natural products augmented intracellular reactive oxygen species (ROS). In this review we will demonstrate the synergistic anticancer effects of a combination of natural products with chemotherapeutic agents or natural products against human HCC and provide new insight into the development of novel combination therapies against HCC.

## 1. Introduction

Hepatocellular carcinoma (HCC) represents the fifth most common cancer and third most common cause of cancer death [1]. Unfortunately, HCC tumors are highly resistant to currently available chemotherapeutic agents. The clinical outcome of HCC treatment remains unsatisfactory. Therefore, new effective and well-tolerated therapy strategies are urgently needed.

The mechanisms underlying the pathogenesis and development of HCC are complex and heterogeneous. They involve multiple cellular signaling pathways, including the Wnt/β-catenin signaling pathway [2], p53 expression [3], Ras/mitogen-activated protein kinase (MAPK) pathway [4], activation of the retinoblastoma protein (pRb) [5], and signal transducer and activator of transcription 3 (STAT3) [6]. These molecular pathways modulate the expression of key genes that are involved in the regulation of cell proliferation, apoptosis, and angiogenesis and that are participants in the processes of induction, progression, and metastasis of hepatic cancer. Thus, each serves as a potential target for novel therapeutics.

Natural products are excellent sources for developing new medications for treating diseases. Recently, the minimal side effects of natural products in anticancer treatments have been recognized. A significant number of drugs that are used to treat cancer are of natural origin [7,8]. The number of natural compounds with anticancer activities that have been discovered and tested *in vitro* and *in vivo* is increasing exponentially. However, many of these natural compounds have failed to gain favor as single-agent anticancer drugs because of a lack of potency. Conventional chemotherapy plays an important role in the treatment of cancers, but clinical limitations exist because of dose-limiting side effects and drug resistance. Therefore, combination treatment of chemotherapeutic agents and natural compounds are considered to be a promising therapeutic strategy with a higher clinical efficacy. Recently, more natural compounds, including genistein, curcumin [9], (−)-epigallocatechin-3-gallate, and resveratrol [10,11], have been recognized as cancer chemopreventive agents because of their anti-carcinogenic activity. They exert their anticancer effects by modulating different cell signaling pathways. Furthermore, we [12,13,14] and other researchers [15,16] have suggested that the combined effect of natural products may improve the effect of treatments against the proliferation of cancer cells. In this review we introduce a combination of natural products with chemotherapeutic agents or natural products and show synergistic anticancer effects against human HCC.

## 2. The Combination of Natural Products with Chemotherapeutic Agents

### 2.1. The Combination of the Natural Product Gambogic Acid with Proteasome Inhibitor MG132 or MG262

Gambogic acid (GA) is a natural product that has been isolated from the gamboge of the *Garcinia hanburyi* tree. Gamboge is a dry resin and has been used as a coloring agent as well as in traditional Chinese medicine for the treatment of human diseases, including indigestion, inflammation, and ulcers [17]. Recent studies have reported that GA has anticancer effects via a variety of cellular signaling effects on different cancer cells, including HCC, gastric and lung carcinoma, breast cancer, and glioma cells [18,19,20,21,22,23,24,25,26]. Zhao *et al.* [27] examined the pharmacological toxicity of GA on the dog cardiovascular and respiratory system and mouse nervous system and showed that GA had no significant side effects on the cardiovascular, respiratory, or central nervous system at higher doses (4 mg/kg/day) than the recommended human doses (25 mg/60 kg/day). These results provided support for clinical applications of this promising natural anticancer agent.

The ubiquitin-proteasome system is responsible for the degradation of most poly-ubiquitinated proteins, including regulatory proteins, that are involved in critical cellular processes, including cell cycle progression, cell development and differentiation, apoptosis, angiogenesis, and cell signaling pathways [28,29,30]. Therefore, targeting the ubiquitin–proteasome pathway has emerged as a rational approach to treat human cancers [31,32]. Bortezomib is the first Food and Drug Administration-approved proteasome inhibitor that has been used as a frontline therapy in refractory multiple myeloma [33].

Huang *et al.* [34] investigated the combined effect of GA and a proteasome inhibitor in human leukemia K562 cells, mouse hepatocarcinoma H22 cells and H22 cell allografts. They reported that the combination of the natural product GA and proteasome inhibitor MG 132 or MG 262 results in a synergistic inhibitory effect on the growth of malignant cells and tumors in allograft animal models [34].

### 2.2. The Combination of Tea Catechins with Doxorubicin

Doxorubicin (DOX) is routinely used as a single drug for the treatment of patients with HCC [35]. It intercalates into DNA, stabilizes the topoisomerase II protein, and causes cell death via inhibition of topoisomerase II and generation of reactive oxygen species and free radicals by redox reactions [36]. Although doxorubicin is an effective antineoplastic agent and has cytotoxic effects, resistance limits its use in chemotherapy [35].

(−)-Epigallocatechin-3-*O*-gallate (EGCG), one of the main polyphenols in green tea, has a variety of physiological and pharmacological effects. EGCG induces apoptosis and inhibits the proliferation of tumor cells [37,38]. EGCG has been extensively studied and has been reported to have chemopreventive effects for many different cancers such as liver, prostate, stomach, esophagus, colon, pancreas, bladder, skin, lung, and breast. EGCG also, has chemopreventive effects in carcinogenesis induced by UV light, chemical agents and genetic aberrations [39].

Liang *et al.* [40] have shown that EGCG also serves as a promising chemosensitizing enhancer for DOX in HCC treatments. Liang *et al.* also showed that catechins inhibit the expression of multidrug resistance 1 (MDR1) mRNA and decrease the levels of P-glycoprotein, a membrane transporter that pumps a wide range of xenobiotics [41], in DOX-resistant HCC cells, suggesting that the administration of DOX in combination with EGCG or epicatechin gallate inhibits P-glycoprotein efflux pump activity and markedly enhances intracellular DOX accumulation [40]. Furthermore, they also found that a therapeutic regimen of EGCG co-treatment with DOX enhanced the antineoplastic efficacy mediated by suppressing autophagy [42]. On the other hand, clinical use of DOX is limited by cumulative cardiotoxicity [43,44]. Saeed *et al.* found that EGCG possesses cardioprotective actions against DOX-induced cardiotoxicity by suppressing oxidative stress, inflammation, and apoptotic signals as well as by activating pro-survival pathways [45]. These studies suggest that the combination of EGCG and DOX is a candidate for improving chemotherapeutic efficacy in HCC treatment.

### 2.3. The Combination of Flavonoids with Conventional Chemotherapeutic Drugs, Cisplatin and 5-Fluorouracil

Zhao *et al.* examined whether the combination of quercetin, a natural flavonoid, with cisplatin, a conventional chemotherapeutic drug, would have synergistic suppressive effects on HCC cells [46].

Quercetin (3,3′,4′,5,7-pentahydroxyflavone), a polyphenolic flavonoid, is abundant in fruits and vegetables. It has been reported to have anti-oxidative, anti-inflammatory, and anticancer effects [47]. Many studies indicate that Quercetin can exert growth-suppressive effects in a variety of types of cancer cells, including esophageal [48], pancreatic [49], colon, and breast cancer cells [50]. In HCC cells, quercetin blocked cell cycle progression at the G1 phase by elevating cyclin-dependent kinase inhibitor p21 and p27 [51]. Tan *et al.* also showed that quercetin induces HCC cell apoptosis by downregulating surviving and bcl-2 [52]. Regarding the toxicity of quercetin, Heinz *et al.* [53] reported that quercetin (0.5 and 1 g/day) did not alter blood leukocytes subsets, granulocyte oxidative burst or phagocytes activity, IL-6, or TNF in healthy females.

Cisplatin (cis-diamminedichloroplatinum(II)) is a commonly used anticancer drug [54]. It exerts its cytotoxic effect primarily by interacting with cellular DNA. Cisplatin binding to DNA alters the structure of the DNA and affects its ability to act as a template in transcription [55]. The effect then triggers apoptotic cell death. However, the clinical use of this drug is limited due to acquired resistance to cisplatin and severe side effects in normal tissues, such as neurotoxicity and acute nephrotoxicity [56].

Thus, development of novel therapeutic strategies is urgently needed.

Zhao *et al.* [46] revealed that the combination of quercetin and cisplatin synergistically inhibits cell growth and triggers apoptosis in HepG2 cells, which involves the alteration of many cell cycle and apoptosis regulators. They suggest that the inclusion of quercetin improves the outcomes of conventional chemotherapy in HCC. However, Li *et al.* [57] examined the effect of the combination of quercetin (20 mg/kg/day) and cisplatin (4 mg/kg/4 days) on a xenograft model of athymic BALB/C-nu nude mice injected with ovarian cancer cells and found that tumors treated with combination of quercetin and cisplatin were significantly heavier than those treated with cisplatin alone. Therefore, they suggested that quercetin treatment supplementation in ovarian cancer patients during chemotherapy may be antagonistic to the cytotoxic effects of chemotherapy [57].

Hu *et al.* [58] investigated the effects of apigenin, the most common phytochemical consumed in the human diet, on enhancing the chemosensitivity of HCC cells and a HCC xenograft model in response to 5-fluorouracil (5-FU).

Apigenin (4′,5,7-trihydroxyflavanone) is available in a wide variety of fruits, vegetables, and herbs [59]. It functions as an inhibitor of specific signal transduction pathways and has been shown to exhibit antitumor activities by inhibiting growth inhibition and inducing cell cycle arrest and apoptosis in many human cancer cells [59,60,61,62].

5-FU, a fluorinated pyrimidine analogue of uracil, is widely used as an anticancer agent in the treatment of gastrointestinal tract, liver, brain, and ovary tumors [63]. Intracellular 5-FU is converted into several active metabolites, such as fluorouridine triphosphate, fluorodeoxyuridine monophosphate, and fluorodeoxyuridine triphosphate [64]. Fluorouridine triphosphate incorporates into RNA and causes the inhibition of pre-rRNA processing, blockage of rRNA post-transcriptional modifications and disruption of pre-mRNA splicing [65]. Fluorodeoxyuridine monophosphate binds to thymidylate synthase and inhibits the conversion of deoxyuridine monophosphate to deoxythymidine monophosphate. Deoxyuridine monophosphate accumulation and fluorouridine triphosphate become misincorporated into DNA, resulting in DNA strand breaks and cell death [66].

Hu *et al.* [58] have shown that sub-toxic concentrations of apigenin (4 µmol/L) significantly enhance the cytotoxicity of 5-FU (100 mg/mL) in HCC cells and that a combined treatment with apigenin (20 mg/kg, five times/week for three weeks) and 5-FU (20 mg/kg for five consecutive days) significantly inhibits the growth of HCC xenograft tumors in nude mice. They demonstrate that apigenin may potentiate the cytotoxicity of 5-FU in HCC by inhibiting reactive oxygen species, followed by a decrease in the mitochondrial membrane potential and activation of the mitochondrial pathway of apoptosis [58].

### 2.4. The Combination of Genistein, a Soy-Derived Isoflavone, with Arsenic Trioxide, a Weak Anticancer Drug for HCC

Jiang *et al.* [67] examined whether genistein synergizes with arsenic trioxide (ATO), which is of limited therapeutic benefit for the treatment of solid tumors.

ATO has been widely employed to treat acute promyelocytic leukemia [68]. The anticancer activity of ATO has also been tested in a variety of solid tumors, including HCC. However, the activity of ATO against solid tumors has not been as effective as in acute promyelocytic leukemia [69].

Genistein, which exhibits multiple biochemical effects, has been shown to inhibit the growth of cancers of the breast, prostate, pancreas, and liver [70,71,72]. Furthermore, a recent study has shown that genistein induces apoptosis of HCC cells by increasing intracellular reactive oxygen species and inducing endoplasmic reticulum stress and mitochondrial injury [73]. Zeng *et al.* [74] studied a phase I human trial of genistein with 40 health subjects and showed that the area under the blood concentration–time curve (AUC) and the plasma concentration of the drug increased linearly when the close was increased from 50 to 100 mg; however, the increase was nonlinear when the dose was increased to 300 mg. Takimoto *et al.* [75] also studied a phase I pharmacokinetic and pharmacodynamics analysis of unconjugated soy isoflavones administered to individuals with cancer and showed that oral administration of soy isoflavones gives a plasma concentration of genistein that has been associated with anti-metastatic activity *in vitro*.

Jiang *et al.* [67] incubated HCC cells (HepG2, Hep 3B, and SK-Hep-1) with ATO, genistein, or ATO + genistein for 72 h and determined cell viability using a Cell Counting Kit-8. Consequently, they found that ATO had little effect on the growth index, as compared with control untreated cells, whereas the combination of ATO and genistein led to a 30–40% inhibition of the growth index compared to the control and calculated values for a coefficient of drug interaction (CDI) based on the principles proposed by Chou and Talalay [76]. CDI is calculated according to the absorbance (MTT assay) of each group as follows; CDI = AB (A × B). AB is the ratio of the combination groups to the control group: A or B is the ratio of the single agent groups to the control group. A CDI value less than, equal to, or >1 indicates that the drugs are synergistic, additive, or antagonistic, respectively. A value <0.7 indicates a significantly synergistic effect [77]. The CDI values for HepG2, Hep3B, and SK-Hep-1 cells treated with genistein and ATO were 0.407, 0.543, and 0.448, respectively, indicating that the two drugs had significantly synergistic effects in inhibiting the viability of HCC cells [63]. They also showed that genistein synergized with a low dose of ATO (2.5 mg/kg) to significantly inhibit the growth of HepG2 tumors in mice. Ma *et al.* [78] confirmed that genistein not only potentiated the proliferation-inhibiting and apoptosis-inducing effect of ATO on human HCC cell lines (HepG2 and Hep3B) *in vitro*, but also dramatically augmented its suppressive effect on both tumor growth and angiogenesis in nude mice at a dose of 50 mg/kg/day.

### 2.5. The Combination of Berberine, an Alkaloid Extracted from Various Plants, with Rapamycin, an Immunosuppressive Agent

Guo *et al.* [79] showed that berberine sensitizes rapamycin-mediated HCC cell death.

Rapamycin was initially characterized as a potent antifungal and was subsequently investigated as an immunosuppressant. It was later shown to exert powerful antiproliferative effects on a wide range of eukaryotic cells, including human tumor cells [80]. Rapamycin revealed the mammalian target of rapamycin (mTOR) signaling pathway, which is important for normal cell and cancer cell growth [81]. In cancer, mTOR is frequently hyperactivated and is a clinically validated target for drug development. A large number of preclinical and clinical studies have demonstrated that the inhibition of the mTOR signaling pathway using rapamycin or rapamycin analogs may be a useful therapeutic strategy for HCC [82,83]. Despite the use of rapamycin as a chemotherapeutic agent, the immunosuppressive effect simultaneously induced by rapamycin is problematic.

Berberine, an alkaloid extracted from *Cortis*, has been studied for its multiple biological and pharmacological activities, including anticancer activity in a variety of human cancer cells [84]. Berberine can inhibit the growth of many cancer cell lines, such as the liver, lung, stomach, colon, skin, esophagus, brain, bones, and breast, by suppressing the growth cycle of tumor cells, inhibiting syntheses of DNA and protein and reducing the activity of topoisomerase. It is also reported that the alkaloid could promote tumor cell apoptosis by regulating apoptotic gene expression and decreasing the transmembrane potential of mitochondria [85]. Chu *et al.* [86] treated SiHa-bearing nude mice with placebo or berberine to verify the *in vivo* antitumor effects of berberine and found that berberine (20 mg/kg) feeding induced a 4.1-fold reduction in tumor weight by day 33 without any apparent signs of toxicity as proven by body-weight monitoring throughout the experiment.

Guo *et al.* [79] hypothesized that the combination of rapamycin and berberine may increase the efficacy of chemotherapy for HCC by synergistically suppressing the mTOR signaling pathway. They found that the combined use of rapamycin and berberine had a synergistic cytotoxic effect. Berberine was observed to maintain the cytotoxic effect of rapamycin on HCC cells at a lower rapamycin concentration. In cells co-treated with berberine and rapamycin, overexpression of CD147 was found to significantly inhibit the downregulation of phosphorylated mTOR expression and decrease cell death. These findings suggest the possibility of developing a novel regimen that is capable of improving HCC therapy and minimizing the immunosuppression associated with rapamycin by decreasing its dose [79].

## 3. The Combination of a Natural Product with a Natural Product

### 3.1. The Combination of *Zizyphus Jujuba* Extract with Green Tea Extract

In China, many people drink jujube tea instead of tea alone. They believe that the combination of *Z. jujuba* with green tea has synergistic effects that enhance immune function. However, the additive or synergistic effect of combining *Z. jujuba* with the extract of green tea on anticancer activity *in vitro* or *in vivo* has not been reported. Huang *et al.* investigated the effect of *Z. jujuba* extract and green tea extract on and their underlying mechanisms of action in HepG2 cells [12,13].

*Z. jujube*, or the Chinese date, is scientifically known as *Zizyphus jujuba* Mill. It is also known as Hongzao or Dazao in China and Natsume in Japan. Believed to have various biological activities, it has been mentioned in the ancient famous Chinese medical book *Sheng Nong Ben Cao Jing* and has been traditionally used in Oriental medicines. For example, in Chinese traditional medicine, its dried fruits are prescribed as an anodyne, anti-tumor, pectoral, refrigerant, sedative, stomachic, styptic, and tonic. In Japan, the extracts of *jujube* are used to treat chronic hepatitis or distress and fullness in the chest and ribs [87].

Japanese people and people throughout the world have consumed green tea beverages for centuries. Epidemiological studies have shown that the consumption of tea is effective in cancer prevention [88,89]. This effect has been attributed to green tea polyphenols, including flavanols, which are commonly known as catechins [90]. In addition, the ability of green tea catechins to improve metabolic abnormalities and reduce body weight has been reported by a number of basic and clinical studies [91,92]. Furthermore, the inhibition of carcinogenesis by tea has been demonstrated in many different animal models such as lung, skin, esophagus, and liver cancer [93,94,95].

One of the authors of this review, Huang *et al.*, found that green tea extract enhances the cytotoxic effect of jujube extract in HepG2 cells and causes cell growth inhibition. The involved mechanisms may be via two pathways. One pathway involves increased p53 and p21 proteins. The increased p21 binds with Cdk2 and prevents Cdk2 from binding to cyclin E, resulting in G1 phase arrest. Decreasing cyclin E levels might also lead to a direct decrease in cyclin E-cdk2 complex levels and cause G1 arrest [12]. Huang *et al.* also showed that the mechanism for the anticancer activity of the combination of jujube extract and green tea extract is via reduction of the expression of APRIL, a proliferation-inducing ligand, and involves upregulation of the p53 and p21 proteins in HepG2 cells [13]. These results suggest that the jujube extract and green tea extract mixture might provide a new drug design to treat hepatocellular carcinoma in the future.

### 3.2. The Combination of Resveratrol with Other Natural Products

Resveratrol is a natural phenol that is produced by several plants and is mainly found in the skin of grapes and red wine. Resveratrol-containing plants, such as *Rheum officinale* Baill and *Polygonum cuspdatum*, have long been used in traditional Chinese medicine [96,97]. In 1997 Jang *et al.* reported that resveratrol use prevents skin cancer development in a mouse model [10]. Many researchers have also suggested that resveratrol administration prevents skin and colon cancer in animal models with artificially induced cancer [98]. Hebber *et al.* [99] treated male and female CD rats with high doses of resveratrol (0.3, 1.0, and 3.0 g/kg/day) for a period of 28 days and studied the dose response using cDNA stress assays coupled with drug-metabolizing enzyme assay. Then they found that at low doses, *i.e.*, 0.3 and 1.0 g/kg/day, there were less significant changes over control rats and at the highest dose (3 g/kg/day) there were changes in gene expression that may be attributed to the toxicity. In addition, many clinical trials on resveratrol in the broad content of inflammation-associated disorder can be found on http://www.clinicaltrials.gov/ (Homepage of A service of the U.S. National Institutes of Health).

Recently, it has been reported that resveratrol has anticancer activity against HCC [100]. The response of HCC to resveratrol includes upregulation of Sirt1 expression [101], increase of JNK and ERK1/2 MAP kinase activity [102], inhibition of VEGF expression [103], and downregulation of cyclin D1 [104]. These molecular mechanisms may underlie the resveratrol-induced apoptosis or self-protection response of the HCC cells.

#### 3.2.1. Synergistic Anticancer Effects of Curcumin and Resveratrol

Curcumin is a polyphenol and major component of the spice turmeric. Turmeric is derived from the rhizome of the Indian plant *Curcuma longa*, which is a member of the Zingiberacae (ginger) family and used in various food preparations. Curcumin inhibits cell proliferation and induces apoptosis in numerous types of cancer cells, including prostate [105], breast [106], colon [107], and liver cancer [108]. The safety, tolerability, and nontoxicity of curcumin at a high dose (8 g/day) are well established by human-clinical trials [109,110]. However, its low bioavailability due to poor absorption and rapid metabolism has been shown to limit its therapeutic efficacy [111]. Therefore, it is necessary to work on improvement strategies.

The combination of curcumin and resveratrol was found to demonstrate a synergistic anticancer effect in colon cancer [112]. In addition, Du *et al.* evaluated the combined effect of curcumin and resveratrol against HCC cells [15]. They demonstrated that the combination treatment of curcumin and resveratrol elicits a synergistic anticancer effect in Hepal-6 HCC cells via extrinsic and intrinsic apoptosis and is associated with reactive oxygen species (ROS) generation and downregulation of X-linked inhibitor of apoptosis protein (XIAP) and survivin, an anti-apoptosis gene. Their study suggested that a combination of curcumin and resveratrol is a promising novel anticancer treatment strategy for liver cancer.

#### 3.2.2. Synergistic Anticancer Effects of Artemisinin and Resveratrol

Artemisinin, a sesquiterpene lactone, is a natural product that is isolated from the plant Artemisia annual or sweet wormwood [113,114]. It contains an endoperoxide moiety that reacts with atomic iron to form cytotoxic free radicals. Artemisinin has been widely used as an antimalarial compound [115]. Artemisinin and its derivatives showed anti-proliferative effects on various tumor cell lines, including cancers of the breast, prostate, colon, and liver [116,117,118]. Artemisinin primarily induces apoptosis via activation of caspase-3 and increasing the Bax/Bcl-2 ratio and polyADP-ribose polymerase [119]. It has also been reported that artemisinin inhibits TNF-α-induced production of proinflammatory cytokines via inhibition of NF-κB and PI3 kinase/Akt signaling pathways [120].

Hou *et al.* studied the *in vivo* antitumor activity of artemisinin in mouse HepG2 and Hep3B xenograft models. In their study, animals were treated with artemisinin at oral doses of 50 and 100 mg/kg/day, when mean tumor mass reached 100 ± 40 mg and artemisinin showed a dose-dependent inhibitory effect on tumor growth [121].

Li *et al.* confirmed that combining artemisinin with resveratrol generated a synergistic effect in an *in vitro* model with HeLa and HepG2 cells and showed that the combination of artemisinin and resveratrol exhibited the strongest effect at the ratio of 1:2 (artemisinin to resveratrol) [16]. Furthermore, the following fluorescent microscopy measurements and cytometry demonstrated that artemisinin and resveratrol effectively inhibited the proliferation of cancer cells and enhanced migration, apoptosis, necrosis and ROS levels. These results suggest that combining artemisinin with resveratrol is a hopeful strategy for a clinical therapy for solid tumors.

#### 3.2.3. Synergistic Anticancer Effects of Matrine and Resveratrol

Matrine, an alkaloid extracted from *Sophora flavescens* Ait, is a natural compound of traditional Chinese medicine and exhibits many biological activities, such as anti-inflammatory [122], anti-virus [123], anti-fibrosis [124], and anti-arrhythmia effects [125], as well as immunosuppression [126]. Recently, some studies showed that matrine had potent anticancer activities by inhibiting proliferation and inducing apoptosis of gastric cancer [127], lung cancer [128], HCC [129], breast cancer [130], and melanoma [131] cells. In addition, Ma *et al.* [132] evaluated an *in vivo* antitumor efficacy of matrine in murine hepatocellular carcinoma H22 inoculated BALB/c mice and showed that seven doses of matrine at 50 mg/kg/dose inhibited 60.7% of tumor growth.

Ou *et al.* examined the effect of the combined treatment of resveratrol and matrine on HepG2 cells and found that the combined treatment significantly enhanced the anti-proliferative effect compared with either agent alone [133]. In addition, resveratrol-induced apoptosis was significantly enhanced by matrine, which could be attributed to the activation of caspase-3 and caspase-9, downregulation of survivin, induction of ROS generation, and disruption of mitochondria membrane potential [133]. These results suggest that the combination treatment of resveratrol and matrine is a promising novel anticancer strategy for liver cancer.

### 3.3. Synergistic Anticancer Effects of Betulinic Acid and Ginsenoside Rh2

Li *et al.* have reported that two natural compounds, ginsenoside Rh2 and betulinic acid, synergistically induce apoptosis in human cervical adenocarcinoma (HeLa), human lung cancer A549, and hepatoma HepG2 cells [134].

Betulinic acid, a pentacyclic triterpenoid, can be directly isolated from various plants that are widespread in the tropics. Betulinic acid was initially reported to be selectively cytotoxic to melanoma cells [135]. It was then demonstrated that betulinic acid exhibited several biological activities, including antibacterial, antimalarial, anti-HIV and anticancer effects. Betulinic acid inhibits cell proliferation and induces apoptosis in numerous types of cancer cells [136]. Fulda *et al.* reported that betulinic acid-induced apoptosis was not associated with the activation of ligand/receptor systems, such as CD95, and did not involve p53. Betulinic acid-induced apoptosis was mediated via direct effects on mitochondria [137] Regarding the *in vivo* anticancer activity of betulinic acid, Damle *et al.* [138] demonstrated the effect of betulinic acid on a MCF-7 human breast adenocarcinoma-induced tumor in athymic nude mice and found that betulinic acid also effectively suppressed growth of the tumors by delaying the development of MCF-7 tumors in a dose-dependent manner.

Ginsenoside Rh2 is isolated from the root of *Panax ginseng* and has been shown to have anticancer effects [139]. It is reported that Ginsenoside Rh2 induces cell death in human hepatoma SK-HEP-1, MCF-7 human breast cancer, human leukemia THP-1, and human lung adenocarcinoma A549 cells.

Ginsenoside Rh2 has been reported to induce apoptosis in a caspase 3,8-dependent manner [140] or by activating cyclin A-cdk2 with caspase 3-mediated cleavage of p21 [141]. Other studies have also demonstrated that Ginsenoside Rh2 inhibits proliferation by inducing the protein expression of p21 and reducing the protein levels of cyclin D, reducing pRB phosphorylation, and inhibiting E2F release [142] or modulating MAP kinase [143] in various cancer cells. Recently, Kim *et al.* have reported that the degree of Ginsenoside Rh2-induced activation of AMP-activated protein kinase correlated with differences in sensitivity to apoptosis in cancer cell lines [144]. However, it is not ideal to use ginsenoside Rh2 singly as a chemotherapeutic agent.

Li *et al.* have shown that two natural compounds, Ginsenoside Rh2 and betulinic acid, synergistically induce apoptosis in human cervical adenocarcinoma (HeLa), human lung cancer A549, and human hepatoma HepG2 cells [134]. They found that co-treatment with Ginsenoside Rh2 and betulinic acid triggered caspase-8 processing and cleavage and sensitized tumor cells by a Bax-dependent mechanism, followed by caspase-9 and -3 processing and apoptosis. These results suggest that co-treatment with Ginsenoside Rh2 and betulinic acid could be a novel strategy to enhance the efficacy of betulinic acid-based therapy.

### 3.4. Synergistic Anticancer Effects of 1′-Acetoxychavicol Acetate and Sodium Butyrate

Kato *et al.* have reported the synergistic effect of 1′-acetoxychavicol acetate and sodium butyrate on the death of human HCC cells [14].

1′-Acetoxychavicol acetate (ACA) naturally occurs in the rhizomes and seeds of *Zingiberaceae* plants, such as *Languas galangal* and *Alpinia galangal.* Southeast Asia residents are traditionally exposed to ACA when using plants as a spice or medicine in everyday life. ACA exhibits chemopreventive effects on chemical-induced tumors in mouse skin and rat oral, colonic, esophageal, and pancreatic tumors [145,146]. ACA also exerts anticancer activity by inducing apoptosis in various tumor cells, such as Ehrlich ascites tumor cells [147], rat and human HCC cells, human colon cancer cells, and human myeloid leukemia cells. Furthermore, we showed that ACA induced apoptosis in Ehrlich ascites tumor cells by decreasing intracellular polyamines and inducing caspase-3 activity [147].

Sodium butyrate has multiple effects on tumor cells cultured *in vitro* by inducing inhibition of cell proliferation and apoptosis [148,149], as well as initiating the differentiation of various carcinoma cells [150,151]. Butyrate also alters the transcription of several genes related to tumor growth and invasiveness and suppresses the growth of tumors implanted in nude mice [152]. Sodium butyrate is an inhibitor of histone deacetylase, which is a class of proteins that can inhibit malignant cell growth *in vitro* and *in vivo*, reverse oncogene-transformed cell morphology, induce apoptosis, and enhance cell differentiation.

Kato *et al.* [14] evaluated the combination of ACA and sodium butyrate on the growth of human HCC HepG2 cells and found that treatment had a synergistic inhibitory effect. The number of HepG2 cells was synergistically decreased via apoptosis induction when the cells were treated with both ACA and sodium butyrate. Furthermore, the intracellular reactive oxygen species (ROS) levels and NADPH oxidase activities were significantly increased in the ACA- and sodium butyrate-treated cells. AMP-activated protein kinase (AMPK), a cellular energy sensor, plays an essential role in controlling processes related to tumor development. The combined treatment of ACA and sodium butyrate significantly induced AMPK phosphorylation. This induction improved when cells were pretreated with catalase [14]. These results suggest that the increase in intracellular ROS is involved in the increase of AMPK phosphorylation. These findings may provide new insight into the development of novel combination therapies against HCC.

## 4. Conclusions

HCC is highly resistant to the currently available chemotherapeutic agents. Therefore, new effective and well-tolerated therapy strategies are needed. This literature review summarizes the current literature on combination treatments with natural products and chemotherapeutic agents or natural products for inhibiting the growth of HCC. As shown in Table 1, a growing body of combination treatments with natural products has been reported to synergistically prevent tumor growth, which has provided new insight into the development of novel combination therapies against HCC. Many combination treatments with natural products can generate intracellular ROS and then induce mitochondrial depolarization and a permeability transition. The formation of ROS could be classified into two general categories: ROS derived from mitochondrial oxygen consumption or ROS that are mitochondrial-independent. It is necessary to elucidate the exact molecular mechanisms for the formation of ROS. In addition, it is important to understand the interaction of these natural products in signaling pathways. Because quercetin has been reported to act as an antagonist to cisplatin, it is also necessary to further examine the effect of the combination treatment of tumor growth in *in vivo* animal models. Then we can respond to a growing demand for testing these natural products in clinical trials, which will be possible after gaining insight into their interactions with exact signaling pathways and the bioavailability as well as the cytotoxicity of these natural products. Furthermore, as with most preclinical leads, positive *in vitro* data does not directly correlate to positive *in vivo* data due to poor solubility and minimal accumulation at the target site, leading to significantly increased systemic toxicity. Regarding this problem, Ling *et al.* [153] have described the interesting report that nanoformulation of natural product facilitated uptake into the tumor, and specifically tumor cells, leading to a further increase in efficacy while mitigating systemic toxicity. Therefore, it is also important to discover and develop more effective therapeutic strategies against HCC.

**Table 1:** Summary of synergistic anticancer activities of natural products in HCC.

Authors [Reference]	Combination Treatments		Comment
	Natural Products	Chemotherapeutic Agents	
Huang H. *et al.* [34]	gambogic acid	proteasome inhibitor (MG132, MG262)	inhibition of growth of cancer cells and tumors in allograft animal model
Liang G. *et al.* [41]	catechins	doxorubicin	Inhibition of P-glycoprotein efflux pump activity
Zhao JL. *et al.* [46]	quercetin	cisplatin	increases in p21 and p53, suppressing cell growth and inducing apoptosis
Hu XY. *et al.* [58]	apigenin	5-fluorouracil	ROS levels ↑, mitochondrial membrane potential ↓
Jiang H. *et al.* [67]	genistein	arsenic trioxide	ROS levels ↑, mitochondrial membrane potential ↓
Guo N. *et al.* [79]	berberine	rapamycin	Synergistic inhibition of the mTOR signaling pathway
	**Natural products**	**Natural products**	
Huang X. *et al.* [12]	*jujube* extract	tea polyphenols	increases in p21 and p53, G1 arrest ↑
Du Q. *et al.* [15]	curcumin	resveratrol	ROS levels ↑, activation of caspase-3, -8 and -9
Li P. *et al.* [16]	artemisinin	resveratrol	ROS levels ↑, apoptosis and necrosis ↑
Ou X. *et al.* [133]	matrine	resveratrol	ROS levels ↑, mitochondrial membrane potential ↓
Li Q. *et al.* [134]	ginsenoside Rh2	betulinic acid	apoptosis through a mitochondria pathway
Kato R. *et al.* [14]	1′-acetoxychavicol acetate	butyrate	ROS levels ↑, increase in the phosphorylation of AMPK
